# Efficacy and Safety of the Ketogenic Diet for Mitochondrial Disease With Epilepsy: A Prospective, Open-labeled, Controlled Study

**DOI:** 10.3389/fneur.2022.880944

**Published:** 2022-08-01

**Authors:** Lijuan Huang, Hua Li, Jianmin Zhong, Liming Yang, Guohong Chen, Dong Wang, Guo Zheng, Hong Han, Xiong Han, Yiqin Long, Xu Wang, Jianmin Liang, Mei Yu, Xiaoyun Shen, Mengke Fan, Fang Fang, Jianxiang Liao, Dan Sun

**Affiliations:** ^1^Department of Neurology, Tongji Medical College, Wuhan Children's Hospital, Huazhong University of Science and Technology, Wuhan, China; ^2^Department of Epilepsy Center, Guangdong 999 Brain Hospital, Guangzhou, China; ^3^Department of Neurology, Jiangxi Provincial Children's Hospital, Nanchang, China; ^4^Department of Neurology, Hunan Provincial Children's Hospital, Changsha, China; ^5^Department of Neurology, Henan Provincial Children's Hospital, Zhengzhou, China; ^6^Department of Neurology, Xi'an Children's Hospital, Xi'an, China; ^7^Department of Neurology, Nanjing Children's Hospital, Nanjing, China; ^8^Department of Neurology, Children's Hospital of Shanxi, Taiyuan, China; ^9^Department of Neurology, Henan Provincial People's Hospital, Zhengzhou, China; ^10^Department of Neurology, Liuzhou Maternal and Child Healthcare Hospital, Liuzhou, China; ^11^Department of Neurology, Changchun Children's Hospital, Changchun, China; ^12^Department of Neurology, The First Bethune Hospital of Jilin University, Changchun, China; ^13^Department of Neurology, Beijing Children's Hospital, Capital Medical University, Beijing, China; ^14^Department of Neurology, Shenzhen Children's Hospital, Shenzhen, China

**Keywords:** mitochondrial diseases, epilepsy, gene, ketogenic diet, MELAS

## Abstract

**Background:**

The ketogenic diet (KD) is increasingly used to treat drug-resistant epilepsy because of its favorable effect on seizure reduction. Patients with mitochondrial diseases tend to experience seizures. Therefore, this study aimed to test the efficacy of the KD on participants with mitochondrial diseases in a controlled trial.

**Methods:**

Participants from fourteen clinical centers who were diagnosed with mitochondrial disease were semi-randomized to either the intervention (KD) or control group. The KD group followed a 3-month KD intervention, while the control group received a 1-month normal diet initially and then a 3-month KD intervention. The primary outcome measure was seizure reduction. Biomarker changes, cognitive impairments, and side effects were also recorded, if available.

**Result:**

A total of 33 participants were assigned to the KD (*n* = 22) and control groups (*n* = 11). In the KD group, 31.8% (7/22) of participants achieved ≥50% seizure reduction after 1 month of diet intervention, which increased to 40.9% (9/22) at 3 months. In the control group, only 18.2% (2/11) of the participants had ≥50% seizure reduction during the normal diet period. After the control group was transferred to the KD, 63.6% (7/11) of participants had >50% seizure reduction, and this rate increased to 72.7% (8/11) at 3 months. The KD also showed high efficacy in participants with mitochondrial encephalopathy, lactic acidosis, and stroke-like episodes (MELAS) or pathogenic variants in mitochondrial DNA (mtDNA) (90% and 93.3% response rates, respectively). The most frequent side effects reported at the 3-month review were vomiting, cold, hyperlipidemia, and bloating.

**Conclusion:**

The KD is a safe and effective therapy for seizure control in mitochondrial diseases, especially MELAS and pathogenic variants of mtDNA. KD intervention can be considered in the management of these patients.

## Introduction

Mitochondrial diseases are a group of heterogeneous disorders caused by dysfunction of adenosine triphosphate (ATP) synthesis and insufficient energy sources. ATP is mainly produced by the mitochondrial respiratory chain and is regarded as a molecular energy source. Thus, mitochondrial dysfunction is more significant in high metabolic organs/tissues, such as the central nervous system (CNS), heart, muscle, liver, and kidney. The CNS is one of the principal organs susceptible to mitochondrial diseases, as it is the latest organ in tissue differentiation, lacks self-repair ability, has a high energy demand and low ATP storage capacity, and is prone to mitochondrial function disorders. The incidence rate of epilepsy in mitochondrial disease ranges from 35 to 60%, and one-third of patients with refractory epilepsy have biochemical evidence of mitochondrial dysfunctions (1).

All types of mitochondrial diseases have impaired ATP production; therefore, patients present with clinical symptoms of energy failure, such as weakness, hypotonia, reduced physical stamina, muscle atrophy, heart failure, and limitation of eye movements with ptosis. Short stature, reduced muscle bulk, deceleration of head growth, and other developmental issues are observed with mitochondrial diseases as well (2). The etiology of mitochondrial disease is primarily related to genetic factors involving mutations in the nuclear DNA (nDNA) and mitochondrial DNA (mtDNA) (3). The mechanisms of epilepsy in mitochondrial diseases may be related to decreased ATP levels, abnormal calcium absorption, energy exhaustion, and energy resource shortage (4–6). Some investigators believe that mitochondrial diseases and epilepsy are closely correlated, creating a vicious cycle (6).

Diagnosis of mitochondrial disease can be confirmed in some patients by the identification of mtDNA mutation with a test of blood sample assessment, and clinical symptoms can be used to infer some disorders. However, many patients require more systemic diagnostic methods, such as family history, blood and/or cerebrospinal fluid lactate concentration, neuroimaging, cardiac evaluation, and gene testing for mtDNA mutations and nDNA mutations (7). Applications of “cocktail treatment” and anti-seizure medications (ASMs) are still mainstream and first-line approaches to treating mitochondrial diseases with epilepsy. Seizures in mitochondria, including generalized tonic-clonic seizures and status epilepticus, can be treated with traditional ASMs, but few of them work well. Moreover, the utilization of traditional ASMs may affect the function of mitochondrial respiratory chain enzymes or have direct toxicity for mitochondria. For instance, valproic acid may impair mitochondria and liver function, cause teratogenesis, and inhibit carnitine absorption. Some studies have reported that the use of valproic acid may lead to severe results like sudden and fatal liver failure (8–11). In summary, it is a challenge to effectively control seizures and enhance energy metabolism in mitochondrial diseases. Therefore, we propose a ketogenic diet (KD) as a possible treatment for mitochondrial diseases with epilepsy.

The KD is a high fat, low carbohydrate, and balanced protein diet, which is used in the treatment of glucose transporter-1 deficiency syndrome, pyruvate dehydrogenase deficiency (PDHD) syndrome, epilepsy, tumors, and some inherited metabolic diseases. In 2015, the KD became a routine treatment for epilepsy in China (12).

The KD simulates the metabolic consequences of fasting, in which the body utilizes fat as an energy source instead of carbohydrates. Some studies have demonstrated that the KD is effective against mitochondrial diseases, such as epilepsy caused by mtDNA and nDNA defects. Recently, reports of gene studies on mitochondrial disease have begun to explain the mechanisms at work. Apart from the direct anticonvulsant function of medium chain triglycerides, other mechanisms include the dysregulation of fat metabolism, downregulation of carbohydrate metabolic genes, improvement of mitochondrial biogenesis, and regulation of anticonvulsant function by peroxisome proliferator-activated receptor-γ (PPAR-γ) and astrocyte metabolism (13). Additionally, PPAR-γ also seems to be involved in the anticonvulsant effect of anticonvulsant drugs (14–16). Therefore, the use of the KD to treat mitochondrial diseases with epilepsy is theoretically feasible.

Lee et al. investigated the effect of the KD in 24 patients with mitochondrial respiratory chain enzyme deficiency who also had refractory epilepsy. They found that 75% of participants had >50% seizure reduction, and half of the participants were seizure-free (17). A retrospective study of KD application in refractory epilepsy, caused by respiratory chain enzyme deficiency, showed that after KD intervention, seven participants (50%) were seizure free with three having no relapse, one having >90% seizure reduction (not seizure free), and three having 50%−90% seizure reduction (18). Some case studies have reported the application of the KD in other mitochondrial diseases: treated patients had satisfactory therapeutic effects and good tolerance for POLG mitochondrial disease, PDHD syndrome, or respiratory chain complex I deficiency due to Otahara syndrome (19–21).

Although some studies have illustrated the safety and efficacy of KD in mitochondrial disease treatment, most of these were case or small-sample studies, and there have been no relevant studies in China. We conducted a multicenter clinical observational study of KD treatment in patients with mitochondrial epilepsy to confirm the efficacy of KD in the treatment of mitochondrial epilepsy. We aim to summarize the conditions of mitochondrial epilepsy treatment with KD in China, further confirm the efficacy and safety of KD, and help improve the quality of life of the patients.

## Methods

### Study Population

This prospective study was approved by the Medical Ethics Committee of the Chinese Clinical trial registry, with a Clinical trial registration No. of ChiCTR1900020789. Nutritionists from multiple centers received uniform training and a uniform case report form was used. Informed consent was signed by each participant before entering this program. Mitochondrial diseases were confirmed using gene diagnosis (pathogenic gene mutations found in mtDNA or nDNA) and abnormal biomarkers (lactose and pyruvate). If the genetic diagnosis showed variants of uncertain significance, clinical features that could match the mitochondrial disease were also employed. Epilepsy conditions were assessed using the International League Against Epilepsy classification (22). Patients who met the following conditions were excluded: those who received KD therapy in the past, or had other inherited metabolic diseases, immunodeficiency, or severe disorders of the digestive, cardiovascular, respiratory, liver, or urinary systems. We planned to recruit 96 patients aged ≤ 16 years who had mitochondrial diseases with epilepsy from 14 clinical centers (listed in Appendix I) between January 2019 and December 2020. However, a total of only 33 participants were enrolled in the study.

### Procedure

Before KD initiation, physicians presented the participants and their families with plans for the study project, ethics profiles, and basic information of KD, and asked if they were willing to be included in the study. Participants and their families signed informed consent forms and underwent enrollment tests and assessments. Dietitians explained the details about the KD to the families, assessed the nutritional status of participants, and registered the participants' profiles. All qualified participants were randomly assigned to one of two groups: a control (regular diet with additional new ASMs) and study (KD with ASMs) group.

The intervention period for the study group (KD group) was 12 weeks, including the baseline period (1 week), titration period (3 weeks), and observation period (8 weeks). There was no change in ASMs' usage during these periods. KD was started with a 2:1 ratio (fat mass to non-fat mass), no liquid limitation, and no fasting. The ketogenic formula was provided by Shenzhen Zeneca Biotechnology Co., Ltd. The participants started KD as either inpatients or outpatients, based on their condition. The dietitian calculated the daily energy requirements of participants according to their age, height, and weight. On the first day, one-third of the total energy was consumed, and food was separated into three or four meals, based on their eating habits. From days 2–4, the energy intake was two-thirds of the total requirement. Blood ketones and glucose were monitored every 6 h during the first 4 days. All side effects (such as hyperketonemia and hypoglycemia) were taken seriously and promptly managed. From day 5, the energy intake was the full daily requirement. During the baseline period, dietitians recorded the participants' condition using an observation table. The first month of KD was named the efficacy observation period, also called the titration period and was the key period of adjustment. Physicians or dietitians adjusted the KD ratio, daily energy intake, mealtime, number of meals, and calories in each meal, according to the condition of the participants. At week 12, when the project was completed, the participants could decide whether to stop or continue KD. If they continued, there was a 36-month diet follow-up period during which physicians, dietitians, and families discussed the ASM adjustment plan and ensured that participants adhered to the KD for better efficacy.

The control group was initiated with a normal diet and adding new ASMs in the first 4 weeks, after which, they followed the KD course for 12 weeks. The KD course in the control group was the same as that in the study group. Essentially, the control group had a study period of 16 weeks: the normal diet period (4 weeks), baseline period (1 week), titration period (3 weeks), and observation period (8 weeks).

Parents could decide to withdraw from the study at any time for any reason such as poor efficacy or tolerance. Physicians could also terminate KD if the participants experienced side effects or exacerbation of diseases.

### Assessment

The study group underwent daily face-to-face (inpatient) or phone call (outpatient) interviews during the baseline period, weekly phone call interviews during the titration period, and phone call interviews every second week during the observation period. Clinical assessments were performed at the hospital at baseline and after four and 12 weeks of the KD in the study group. The control group underwent clinical assessments at baseline, at week four of regular diet, and after 4 and 12 weeks post KD initiation (weeks 8 and 16 of the study period). Baseline assessments included laboratory tests, nutrition assessments, family interviews, and other tests, if needed, such as growth scales, magnetic resonance imaging (MRI), electroencephalogram (EEG), and biopsy.

The primary outcomes during the study period were efficacy and biomarker levels. Efficacy was classified into four levels: seizure-free, 90–99% reduction, ≥50– <90% reduction, and <50% reduction in seizures. The decrease in the percentages were calculated from seizure records between the baseline and the study period. Only ≥50% reduction in seizure frequency lasting >4 weeks was considered effective. Biomarkers included lactose, pyruvate variation, and blood pH levels, which were measured at the clinical visit assessment. Other outcomes included improvements in EEG, MRI, and quality of life. Cognition, behavior, and growth conditions were evaluated using the Gesell Scale or Wechsler Intelligence Scale for Children. Safety indices included the incidence rate of hypoglycemia, vomiting, diarrhea, constipation, high cholesterol, lithiasis, and other side effects; biochemical monitoring of lipid and carbohydrate metabolism; and disease-related tests such as blood gas analysis and type B ultrasound. Participants who did not continue in this project were included in the safety analysis but not the efficacy analysis. Nutrition indices were assessed at baseline and during the study period, and included weight, height, body mass index, body content, serum protein, and hemoglobin.

### Data Analysis

Data analyses were conducted using the SPSS software (version 26.0). Sex, course length, drug usage, and seizure frequency between the KD and control groups at baseline were analyzed using the Mann-Whitney U test. Age differences between the groups was analyzed using an independent *t*-test. The Fisher's exact test was used to assess differences in response rates among the KD group (1 month KD) and the control group (1 month drug therapy), and the KD and control groups after 1 and 3 months of diet intervention. McNemar's Test was used to analyze the control group before and after drug therapy and KD therapy. Responders were classified as effective (seizure reduction ≥50 to <90%), highly effective (seizure reduction ≥90% to <100%), and seizure-free (seizure reduction 100%).

## Results

### Baseline Characteristics

**Figure 1:**
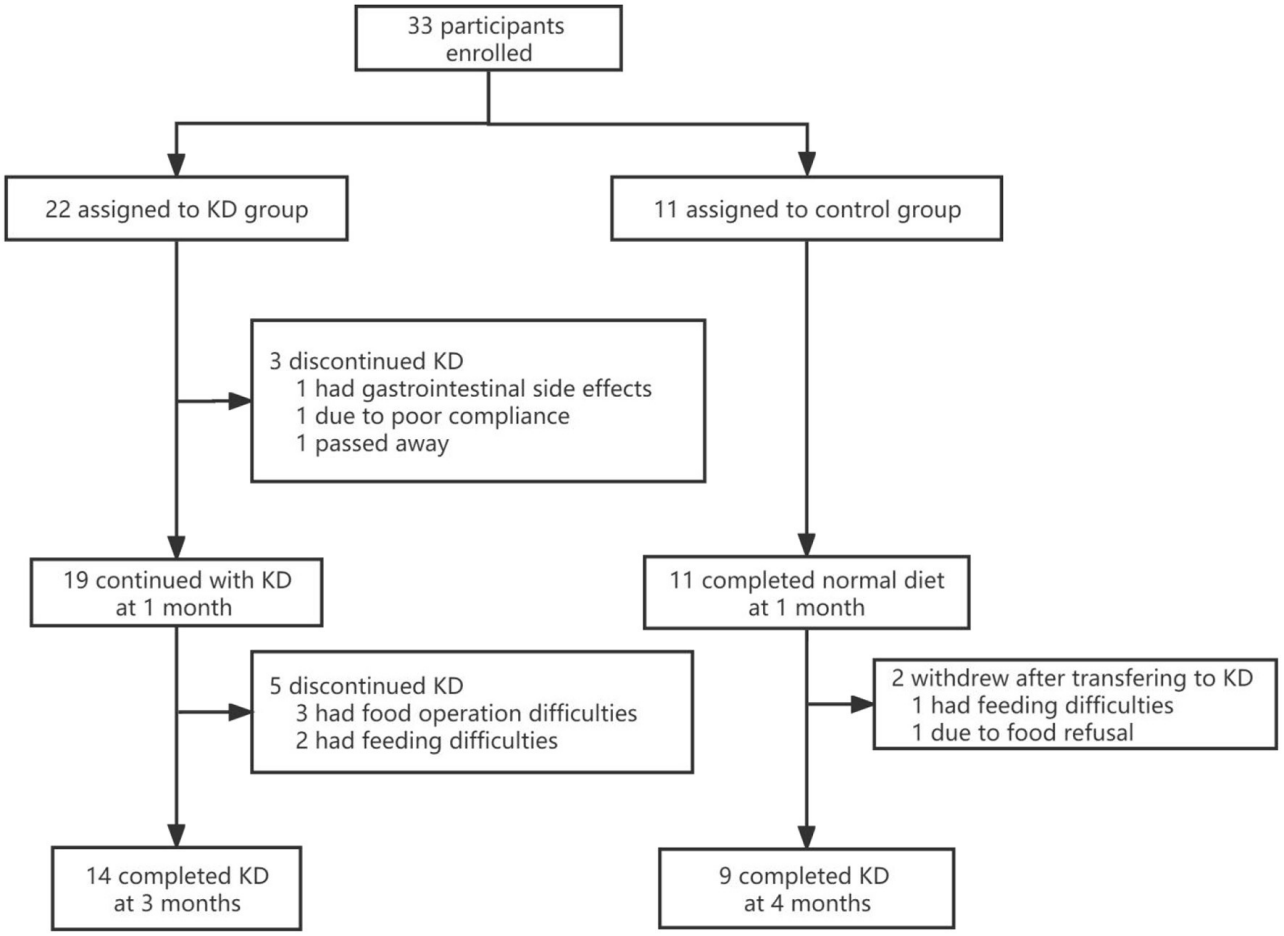
Trial flowchart. KD, ketogenic diet.

A total of 33 participants from 14 clinical centers were enrolled in the study. Participants were semi-randomly assigned to the KD and control groups, with 22 and 11 in each group, respectively. No significant differences in sex, age, length of epilepsy course, number of drugs used, or seizure frequency were found between the KD and control groups (*p*>0.05). Ten individuals withdrew from the study (Figure 1). The baseline demographic characteristics and other systemic symptoms of the patients in the two groups are shown in Table 1.

**Table 1:** Characteristics of participants at baseline in two groups.

**Variables**	**KD group (*n* = 22)**	**Control group (*n* = 11)**	***p* value^**a**^**
Male/Female	13/9	8/3	0.534^a^
Median age (months) (min-max, SD)	79 (9–215, 60.6)	76 (5–159, 51.4)	0.476^b^
Median length of the disease course (months) (min-max, SD)	14.5 (2–140, 35.6)	11(3–65, 17.3)	0.336^a^
Median number of used drugs (min-max, SD)	2 (0–5, 1.7)	1 (1–3, 0.7)	0.155^a^
Median of seizure frequency per month (min- max, SD)	180 (0.25-900, 240.5)	50 (0.26–240, 77.1)	0.072^a^
Disease diagnosis			
MELAS	9	5	
Suspected MELAS	0	1	
MERRF	1	0	
PDHD	0	1	
Leigh	1	0	
COQ10D7 with epilepsy	1	0	
Uncategorized	10	4	
Hyperlactacidemia			
YES	15	6	
NO	3	2	
Unavailable	4	3	
Stroke-like episodes			
YES	9	5	
NO	13	6	
Cognitive evaluation			
Normal	2	1	
Mild intellectual disability	2	2	
Severe intellectual disability	1	0	
Unavailable	17	8	
Gene mutation			
Mitochondrial DNA (mtDNA)			
m.3243 A>G	10	6	
m.3697 G>A	2		
m.8344 A>G	2		
m.3243 A>G+m.3271 T>A		1	
m.127720 A>G		1	
Nuclear DNA (nDNA)			
*COQ4*	2		
*POLG*	2		
*ATP6*		1	
*FARS2*	1		
*FOXRED1*	1		
*PDHB*		1	
*QARS*	1		
*TPK1*		1	
*TRIT1*	1		

*KD, ketogenic diet; MELAS, stroke-like episodes; MERRF, myoclonus epilepsy with ragged-red fibers syndrome; PDHD, pyruvate dehydrogenase deficiency syndrome; COQ10D7 with epilepsy primary coenzyme Q10 deficiency-7*.

*^a^Mann-Whitney U test; ^b^t-test*.

Gene mutations were reported in all participants with mutations in mtDNA reported in 22, and nDNA mutations reported in 11. In this study, m.3243 A>G was the most common pathogenic mtDNA variant, although other gene mutations were also present (Table 1). Most participants were diagnosed with mitochondrial encephalopathy, lactic acidosis, stroke-like episodes (MELAS), and uncategorized mitochondrial diseases with epilepsy (Table 1).

### Efficacy of KD

**Figure 2:**
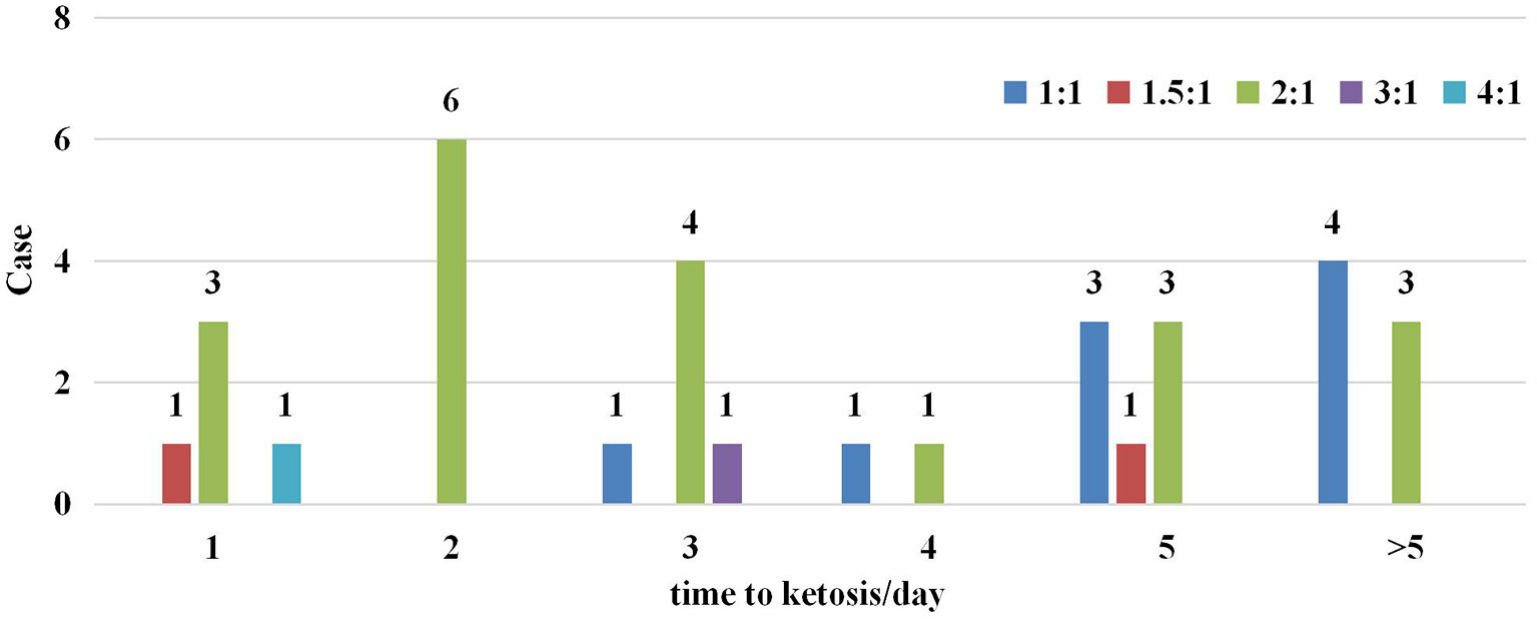
Start ratio of ketogenic diet and time to ketosis in the enrolled cohort.

Five different KD ratios were used in this trial, ranging from 1:1 to 4:1 (Figure 2). A ratio of 2:1 was most common (20/33), only three participants were initiated with a KD ratio higher than 2:1. Overall, 78.8% (26/33) of participants reached ketosis (defined as >2 mmol/L of blood ketones) within 5 days of starting the KD in Figure 2.

In the KD group, 31.8% (7/22) of participants achieved ≥50% reduction in seizure frequency after 1 month: one was seizure-free, one experienced 90%−100% reduction, and five had 50%−90% reduction. At 3 months, two more participants had ≥50% reduction, the efficacy increased to 40.9% (9/22), and two were seizure-free (9.1%). In the control group, only 18.2% (2/11) of participants had ≥50% reduction in seizure frequency with 1 month of general diet plus new medications. However, the seizure condition improved once the participants transferred to KD. One month after the transition to KD, 63.6% (7/11) of the participants in the control group had ≥50% seizure reduction, and this rate increased to 72.7% (8/11) after 3 months (*p* > 0.05). Further 27.3% (3/11) of the participants in the control group were seizure-free. Based on the data analysis, there was no significant difference when comparing the additional drugs treatment with KD intervention at 1 month (*p* > 0.05), however, there was a significant difference between the KD intervention at 3 months and the drugs treatment at 1 month (*p* < 0.05) in the control group. Therefore, these seizure improvements were independent of drug adjustment with a general diet. Additional details are presented in Table 2.

**Table 2:** Seizure frequency reduction of admitted participants.

	**1 month of KD**		**1 month of general diet**	***P* value**
	**A: KD group (*n* = 22)**	B: Control group*(*n* = 11)	**C: Control group (*n* = 11)**	
≥ 50% reduction	7 (31.8%)	7 (63.6%)	2 (18.2%)	**p1 = 0.136^a^, p2 = 0.681^a^, p3 = 0.063^b^
Seizure free	1 (4.5%)	1 (9.1%)	0 (0)	
90%-100% reduction	1 (4.5%)	1 (9.1%)	0 (0)	
50%-90% reduction	5 (22.7%)	5 (45.5%)	2 (18.2%)	
	**3 months of KD intervention**		
	**KD group (*n* = 22)**		Control group*(n=11)
≥ 50% reduction	9 (40.9%)		8 (72.7%)	**p4 = 0.141^a^, p5 = 1.000^b^, p6 = 0.031^b^
Seizure free	2 (9.1%)		3 (27.3%)	
90%-100% reduction	1 (4.5%)		2 (18.2%)
50%-90% reduction	6 (27.3%)		3 (27.3%)

^a^*Fisher's exact test; ^b^McNemar's test*.

*Patients had transferred from general diet to KD (ketogenic diet).

**p1, compare between A and B; p2, compare between A and C; p3, compare between B and C.

*P4, compare between KD group and control group after 3months; P5, compare between B and control group after 3months of KD.*

*P6, compare between control group after 3months of KD and C*.

Some participants experienced favorable biomarker changes and cognitive improvements at 3 months. The clinical information and biochemical examination of the seizure-free cases were listed in Supplementary Table 1. The EEG of one seizure-free participant in KD group returned to normal, and his lactic acid reduced by 5.38 mmol/L (7.64 mmol/L-2.26 mmol/L) compared to that in the screening period. Minor changes were noted in their EEGs of other participants. The lactic acid of two seizure-free participants in the control group reduced by 3.1 mmol/L (7.4 mmol/L-4.3 mmol/L) and 5.7 mmol/L (8.86 mmol/L-3.16 mmol/L). Cognitive evaluations of the seizure-free participants from KD and control group were all unchanged. Six patients had a mean reduction of 2.94 mmol/L of lactic acid. Pyruvate in two patients was reduced by 22.3 and 34.9 mmol/L respectively. One patient's laboratory finding showed a decrease in blood ammonia of 48.2 mmol/L.

### Analysis of the Correlation Between Genomics and Efficacy of KD

Twenty-three participants completed the study, and 17 (73.9%) had ≥50% seizure reduction. Table 3 shows the genetic conditions of the participants who completed the trial. Fifteen had mitochondrial DNA mutations and eight had nDNA mutations. Participants with mtDNA mutations responded remarkably to KD (93.3%, 14/15), nine of whom had the pathogenic variant m.3243 A>G. Participants with nDNA mutation responded less favorably to KD, with an effective rate of only 37.5% (3/8). KD also seemed to be quite effective in MELAS, as nine of 10 participants (including one with suspected MELAS) experienced ≥50% seizure reduction (Table 3).

**Table 3:** Disease diagnosis and Gene diagnosis of completed patients and effective participants.

**Variables**	**Completed participants (n=23)**	**Effective participants (*n* = 17)**
Disease diagnosis		
MELAS	9	8
Suspected MELAS	1	1
PDHD	1	1
Leigh	1	0
Uncategorized	11	7
Gene mutation		
mtDNA Gene mutation	15	14 (93.3%)*
m.3243 A>G	10	9
m.3697 G>A	2	2
m.8344 A>G	1	1
m.3243 A>G+m.3271 T>A	1	1
m.127720 A>G	1	1
nDNA Gene mutation	8	3 (37.5%)*
*COQ4*	1	1
*POLG*	2	1
*FOXRED1*	1	0
*PDHB*	1	1
*QARS*	1	0
*TPK1*	1	0
*TRIT1*	1	0

*The level of m.3243 A>G heteroplasmy in effective participants:*.

*40.55%, 98.79%, 76.4%, 100%, 79.62%, 78.40%, 73.71%, Unavailable (2).*

*MELAS, stroke-like episodes; PDHD, pyruvate dehydrogenase deficiency syndrome;*

*Effective Rate.

### Adherence and Side Effects

Nine participants withdrew from the study, and one died. Seven were from the KD group: two withdrew before 1 month due to gastrointestinal side effects (1) and poor compliance (1), while five withdrew after 1 month due to food preparation difficulties (3) and apocleisis (2). Two patients in the control group withdrew after transferring to KD: one because of feeding difficulties and food refusal, and one had unsatisfactory efficacy. Few side effects were seen in the participants who completed the trial, as shown in Table 4.

**Table 4:** Side effects condition of trial-completed participants.

**Side effects**	**Number of participants**	**Incidence**
Vomiting	3	13.0%
Cold	1	4.3%
Bloating	1	4.3%
Sum	5	21.7%

## Discussion

Our results showed the benefits of KD in mitochondrial disease treatment. Few studies have discussed the efficacy of KD in the treatment of mitochondrial diseases. A recent systematic review reported that 7/8 patients with mitochondrial diseases responded to KD treatment (5 seizure-free, 1 reduction of seizures, and 1 control of status epilepticus) (23). Two other studies with mitochondrial respiratory chain defects in epilepsy patients found that 71–75% of participants experienced >50% seizure reduction after KD intervention (17, 18). The response rates of mitochondrial diseases in this study were similar to those in refractory epilepsy studies, in which response rates for the KD and control groups were 35–56.1% and 6–18.2%, respectively (24–31). In addition, the International League against Epilepsy (ILAE) also attests to considerable response rates (70%) of KD to epilepsy and recommended this therapy in its guidelines (32). On comparing our results to previous studies and ILAE guidelines, with the 76% (19/25) response rate of trial-completed participants, it is reasonable to state that KD could effectively improve seizure conditions of mitochondrial diseases with epilepsy.

Surprisingly, the response rate of the KD group seemed to be lower (although this was not statistically significant) than that of the control group who had changed to KD after 1 month of the control period. However, the explanations for this could vary. First, eight participants in the KD group withdrew, while only two withdrew from the control group after changing to KD. A high withdrawal rate certainly influenced response rates. Second, although most participants in both groups started with a 2:1 ratio diet, more participants changed to a lower ratio in the KD group after dietary adjustment. Some studies have noted that the classic KD with a higher ratio may lead to better seizure control (33). However, other studies have indicated that there is no difference in efficacy between high and low ratios (34). The relationship between the ketogenic ratio and treatment efficacy remains controversial. Another plausible reason for the difference that we reported is participant bias. Even though statistical analysis found no difference in seizure frequency between groups at baseline, it seemed that participants in the KD group had relatively more seizures.

Other than reduction in seizure frequency, some participants experienced favorable biomarker changes and cognitive improvements at 3 months. Similar biomarker changes have also been demonstrated in studies of mitochondrial disease (35, 36). The Neuropsychological Development Scale (0–6 years old) and Wechsler Scale showed that three participants had a development quotient and mental age increase, or total score improvement. The results of cognition improvement in our study were not as good as those from epilepsy treatment (37, 38), probably because only a few participants underwent cognitive assessment.

MELAS is a maternally inherited multisystemic disorder caused by mutations in mitochondrial DNA and is the second largest subgroup of diseases in this study. The relationship between certain gene mutations and MELAS has been extensively discussed. One study showed a high consistency between MELAS and m.3243 A>G variants (39). According to that study, the m.3243 A>G variant in the mitochondrial tRNA gene was present in 21/23 MELAS participants, 11/11 oligosymptomatic relatives, and 12/14 asymptomatic relatives, but not in 5/50 mitochondrial disease (39). Another study indicated that gene variants were found in 26/31 participants with MELAS, but only in 1/29 participants with CPEO (chronic progressive external ophthalmoplegia), 0/5 participants with MERRF (myoclonus epilepsy with ragged-red fibers), and 0/50 control participants (40). In general, more than 80% of participants with MELAS have the m.3243 A>G variant, with m.3271T>C, the second most common mutation, found in 10% of participants (41, 42). In our study, all participants with MELAS were diagnosed with the m.3243 A>G mutation. In addition to the m.3243 A>G variant, some other gene mutations presented in this study were also found to be related to mitochondrial diseases in previous studies, such as m.3697G>A and m.8344A>G (43–45). To the best of our knowledge, mutation of m.127720 A>G and m.15596 G>A, have not previously been discussed and our study is the first to relate them to a particular disease.

In our study, KD was found to be highly effective (89%) in participants with MELAS. However, the relationship between MELAS and KD has not been studied extensively. In a cellular model, exposure of neuronal cells to ketone bodies increased ATP synthesis, improved mitochondrial metabolism, and restored the activity and stability of complex I, which is impaired in MELAS (46). In a case study, a 22-year-old female patient's seizures were unaffected by the addition of new anti-epileptic medications; however, after receiving a modified KD, she experienced an improvement in seizure control and a decrease in stroke-like episodes (47). From a genetic perspective, KD strongly effects mitochondrial DNA mutations, especially the m.3243 A>G variant, which was the largest gene mutation subgroup. An earlier study indicated that a severe m.3243 A > G mutation might impair fuel catabolism and cause MELAS (48). Some studies have proposed that KD may induce electron transport chain subunit mRNA to increase mitochondrial biogenesis. This results in increased ATP levels, leading to increased neuronal “energy reserves,” allowing neurons to withstand metabolic challenges and stabilize neuronal membrane potentials (49, 50). KD appeared to be effective for other mtDNA mutations in this study, including m.3697 G>A, m.8344 A>G, m.3243 A>G+m.3271 T>A, m.127720 A>G. The response rate of nDNA mutations was much lower, as only four participants with *COQ4, POLG*, and *PDHB* variants were participants. In nDNA-related research, low glycemic index treatment (LGIT) was found to be effective in treating mitochondrial epilepsy with *POLG1* gene mutation. In the report, the visual aura and aphasia could not be resolved in an adult participant with three ASMs. After the introduction of the LGIT, the headaches, aphasia, and visual aura progressively improved and disappeared. The participant improved and returned home two weeks later, and the seizures disappeared (20).

Ten participants withdrew and 7/10 reported poor compliance. Most of these had trouble with food operations and feeding. The high-fat and low-carbohydrate food composition was quite different from the regular diet, especially in China, where carbohydrate rich foods such as rice are still the predominant component in a meal. Thus, education and training for participants or caregivers prior to dietary intervention is important. Some researchers believe that new therapies are urgently needed to broaden the management options and improve the prognosis of patients (51), thus, tools of e-health management and recipe software could have increased the treatment compliance (52, 53). Additionally, correct management of the KD in patients with refractory epilepsy is important from the beginning to avoid side effects (54). One participant died during the study period. Epilepsy was noticed in this participant 2.5 months after birth, and he was diagnosed with *COQ10D7*. His eating ability was poor; therefore, constant tube feeding was used. At 11-months of age, he was admitted to the KD group and passed away 1 month later. He had normal stools and marked seizure improvement, but experienced vomiting. No other medical condition was present. The cause of death was unknown and may have been related to sleep apnea. According to previous reports, respiratory distress, cerebellar atrophy, and lactic acidosis are common symptoms of *COQ10D7*.

In total, seven participants experienced side effects during the intervention, with five reporting gastrointestinal (GI) side effects, including vomiting, bloating and GI disturbance. This is similar to other studies (26, 55–57) showing that approximately 30%−40% of participants had GI side effects, but our study showed a lower incidence. This difference could account for the relatively low ketogenic ratio observed in our study. One participant reported hyperlipidemia, which is not unusual in KD, but some studies have found that long-term KD intervention often keeps the serum lipid profile within normal limits (58, 59).It is important to manage side effects properly to encourage patients to remain on the KD.

To the best of our knowledge, this is the first prospective controlled study to discuss the efficacy of KD in the treatment of mitochondrial diseases with epilepsy. The larger sample size in this study made the therapeutic effects of KD on mitochondrial diseases more credible. Detailed genetic information for each patient also confirmed that KD was more efficient in MELAS and mtDNA mutations, especially for the m.3243 A>G pathogenic variant. However, our study has some limitations. The first limitation was the brief period of the normal diet in the control group, however a full 3-month period without dietary treatment may not be acceptable to parents. The second limitation is the unbalanced number of participants in the KD and control groups. Third, the use of parental seizure records might have introduced subjective errors such as overestimation or underestimation of seizure numbers. The combined utilization of subject records and EEGs may improve the scientific weight. Long-term blind trials are suggested for further studies.

## Conclusion

Our study showed that KD is effective and can be included in the management of patients with mitochondrial disease and epilepsy. Gene reports indicate that KD is more efficient for MELAS and mtDNA mutations, especially the m.3243 A>G pathogenic variant. The side effects are considerable but should not be excessive. Most side effects were easily manageable, resulting in a few patients withdrawing from diet intervention.

## Data Availability Statement

The original contributions presented in the study are included in the article/Supplementary Materials, further inquiries can be directed to the corresponding authors.

## Ethics Statement

The studies involving human participants were reviewed and approved by the Medical Ethics Committee of Chinese Clinical trial registry, with a Clinical trial registration No. of ChiCTR1900020789. Written informed consent to participate in this study was provided by the participants' legal guardian/next of kin.

## Author Contributions

DS, FF, JL, and LH designed the study and revised the manuscript accordingly. The other authors participated in the study and provided case information. MY, MF, and DS drafted the manuscript. All authors contributed to the article and approved the submitted version.

## Funding

This study was supported by the Fund of Futang Research Center of Pediatric Development (No.FTCSF-2018-05), the Fund of Shenzhen Zeneca Biotechnology Co., Ltd (No.20181208), and China Association Against Epilepsy (CAAE) Research Fund—Qitong Fund (No.CJ-B-2021-20). Shenzhen Zeneca Biotechnology Co., LTD. was not involved in the study design, collection, analysis, interpretation of data, the writing of this article or the decision to submit it for publication.

## Conflict of Interest

The authors declare that the research was conducted in the absence of any commercial or financial relationships that could be construed as a potential conflict of interest.

## Publisher's Note

All claims expressed in this article are solely those of the authors and do not necessarily represent those of their affiliated organizations, or those of the publisher, the editors and the reviewers. Any product that may be evaluated in this article, or claim that may be made by its manufacturer, is not guaranteed or endorsed by the publisher.

## Abbreviations

ASM: anti-seizure medication
ATP: adenosine triphosphate
CNS: central nervous system
EEG: electroencephalogram
GI: gastrointestinal
KD: ketogenic diet
MELAS: mitochondrial encephalopathy, lactic acidosis, and stroke-like episodes
MRI: magnetic resonance imaging
mtDNA: mitochondrial DNA
nDNA: nuclear DNA
PPAR-γ: peroxisome proliferator-activated receptor-γ.
