# Supplementary material for: Efficacy and Safety of the Ketogenic Diet for Mitochondrial Disease With Epilepsy: A Prospective, Open-labeled, Controlled Study
Source: Front Neurol. 2022 Aug 1;13:880944. doi: 10.3389/fneur.2022.880944 (PMC9377015; doi:10.3389/fneur.2022.880944)
Supplement: Supplementary file 1 [file Table_1.docx]

Efficacy and Safety of the Ketogenic Diet for Mitochondrial Disease with Epilepsy: A Prospective, Open-labeled, Controlled Study

Supplementary Table 1. Clinical information and biochemical indicators of seizure-free cases

| Group | No. | The baseline period | | | During control period | | | After KD | | |
| --- | --- | --- | --- | --- | --- | --- | --- | --- | --- | --- |
|  |  | EEG | Lactic acid level (mmol/L) | Cognitive  evaluation | EEG | Lactic acid level (mmol/L) | Cognitive evaluation | EEG | Lactic acid level (mmol/L) | Cognitive  evaluation |
| KD  group | 1# | Right posterior head spikes are released with slow wave | 7.64 | Normal | — | — | — | Normal | 2.26 | Normal |
|  | 2# | Slow wave increased in the right hemisphere; Large low to medium amplitude spikes-slow wave on the right posterior head, significant on the right posterior head. | 1.84 | Mild intellectual disability | — | — | — | Slow waves increased in both hemispheres | 1.99 | Mild intellectual disability (Memory loss) |
| Control  group | 1# | In the wake period, a little more medium-high wave waves with amplitude of 2-4Hz are generated in bilateral parietal, occipital and middle and posterior temporal regions, which can be generalized. | 7.40 | Mild developmental delay | Unavailable | 6.90 | Mild developmental delay | Unavailable | 4.30 | Mild developmental delay |
|  | 2# | In waking and sleeping period, the middle and high amplitude spiky wave and 3-4Hz spiky slow wave are issued in multiple quantities in bilateral occipital and posterior temporal regions | 8.86 | Unavailable | Unavailable | 8.63 | Extremely severe intellectual disability | Unavailable | 3.16 | Extremely severe intellectual disability |
|  | 3# | A large number of irregular sharp slow wave in the right posterior occipital area during waking period, slow wave or sharp wave emission. | Unavailable | Unavailable | Unavailable | 7.35 | Unavailable | Unavailable | 4.00 | Moderate intellectual disability |
